# Multiple Mechanisms of Anti-Cancer Effects Exerted by Astaxanthin

**DOI:** 10.3390/md13074310

**Published:** 2015-07-14

**Authors:** Li Zhang, Handong Wang

**Affiliations:** Department of Neurosurgery, Jinling Hospital, School of Medicine, Nanjing University, Nanjing 210002, Jiangsu Province, China; E-Mail: zhangli2007js@126.com

**Keywords:** astaxanthin, cancer, molecular targets

## Abstract

Astaxanthin (ATX) is a xanthophyll carotenoid which has been approved by the United States Food and Drug Administration (USFDA) as food colorant in animal and fish feed. It is widely found in algae and aquatic animals and has powerful anti-oxidative activity. Previous studies have revealed that ATX, with its anti-oxidative property, is beneficial as a therapeutic agent for various diseases without any side effects or toxicity. In addition, ATX also shows preclinical anti-tumor efficacy both *in vivo* and *in vitro* in various cancer models. Several researches have deciphered that ATX exerts its anti-proliferative, anti-apoptosis and anti-invasion influence via different molecules and pathways including signal transducer and activator of transcription 3 (STAT3), nuclear factor kappa-light-chain-enhancer of activated B cells (NF-κB) and peroxisome proliferator-activated receptor gamma (PPARγ). Hence, ATX shows great promise as chemotherapeutic agents in cancer. Here, we review the rapidly advancing field of ATX in cancer therapy as well as some molecular targets of ATX.

## 1. Introduction

**Figure 1:**
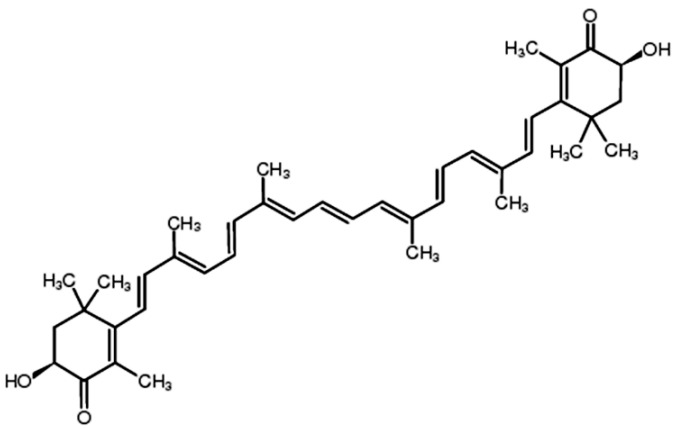
Chemical structure of ATX.

Astaxanthin (ATX), one of the most common carotenoids, is widely distributed in the red pigment of shrimp, salmon, crab and asteroidean [1,2]. In 1987, ATX was approved by the United States Food and Drug Administration (USFDA) as a feed additive for use in the aquaculture industry. And in 1999, it was approved for use as a dietary supplement (nutraceutical) [3]. ATX shows more powerful anti-oxidative property than other carotenoids, such as canthaxanthin, lutein, zeaxanthin and β-carotene [4]. The two oxygenated groups on each ring structure is responsible for its enhanced anti-oxidant features (Figure 1) [3]. It has been suggested that ATX could protect against neurotoxin or oxidative stress-induced damage both *in vivo* and *in vitro* [5,6,7]. Previous researches have used ATX as an anti-oxidant therapeutic agent in models of brain injury [8,9,10] and cardiovascular disease [11,12]. Furthermore, at least 8 clinical studies have been conducted in cardiovascular disease to assess the dosing, bioavailability and safety of ATX [13]. Notably, no significant side effects of ATX have been reported so far. In addition to its potent anti-oxidative effects, evidence suggests that ATX has anti-cancer efficacy in multiple types of cancer, including oral cancer [14], bladder carcinogenesis [15], colon carcinogenesis [16,17], leukemia [18] and hepatocellular carcinoma [19,20]. The anti-cancer effects of ATX are reportedly attributed to its effects on the pathological process of cancer cells through a variety of pathways including apoptosis, inflammation and cell junction. In this review, we describe the latest progress of ATX in cancer therapy (Table 1).

**Table 1:** Effects of ATX on cancers.

Cancers	*In Vitro*/*In Vivo*	Molecular Targets	Functions
Oral cancer	*In vivo*	JAK-2/STAT-3, NF-κB, ERK, AKT (PKB)	Abrogate cell proliferation, invasion and angiogenesis, induce intrinsic apoptosis
Bladder carcinogenesis	*In vivo*	/	Reduce the incidence of cancer and suppression of cell proliferation
Colon carcinogenesis	Both	NF-κB, ERK, JNK, p38, AKT	Inhibite cell growth, invasion and inflammation, induce apoptosis, arrest cell cycle progression
Leukemia	*In vitro*	PPARγ, Nrf2	Decreased cell viability, induce apoptosis and interfere with cell cycle progression
Hepatocellular carcinoma	Both	JAK-1/STAT-3	Attenuate cell proliferation and invasion, induce mitochondria-mediated apoptosis
Lung cancer	*In vitro*	/	Inhibit cell proliferation
Breast cancer	*In vitro*	/	Suppress cell proliferation

JAK: Janus kinase; STAT: Signal transducers and activators of transcription; NF-κB: Nuclear factor kappa-light-chain-enhancer of activated B cells; ERK: Extracellular signal-regulated kinaes; JNK: c-Jun *N*-terminal kinases; PKB: protein Kinase B; PPARγ: Peroxisome proliferator-activated receptor gamma; Nrf2: NF-E2-related factor 2.

## 2. Anti-Cancer Effects of ATX

### 2.1. Anti-Proliferation of Cells

Tumor formation is characterized by rapid proliferation of cancer cells. Cancer cells proliferate promotes its invasion, migrate and adhere to target tissue. These steps allow the tumor cell to obtain metastatic phenotype. Cell proliferation depends on the signals transmitted by growth factors and adhesion proteins [21] and is usually regulated by signaling pathway such as mitogen-activated protein kinase (MAPK) and phosphatidylinositide 3-kinases (PI3K) cascades [22,23,24,25]. The processes of proliferation and further invasion, migration and adhesion require the rearrangement of actin cytoskeleton. It involves the release of pre-existing cell-matrix contacts and formation of new integrin substratum contacts [26]. The effect of ATX on cell proliferation in cancer cells has been explored by many researchers. Song *et al.* [19] have observed the anti-proliferative effect of ATX against CBRH-7919 (human hepatoma), SHZ-88 (rat breast) and Lewis (mouse lung) cells. They reported a strong correlation between ATX concentration and anti-proliferative effect on these cells at 24 h. However, of these cells, CBRH-7919 was the most sensitive cell line to ATX with an IC_50_ value of 39 μM. In a separate study, Zhang *et al.* [18] compared the growth inhibitory effect of ATX with other carotenoids such as β-carotene, capsanthin and bixin on K562 leukemia cells. They found that when K562 cells were treated with low concentrations of carotenoids (5 and 10 μM), ATX was the most effective to inhibit cell growth among the four kinds of carotenoids, followed by bixin, β-carotene and capsanthin in order. In addition, ATX was shown to impede proliferation in a hamster model of oral cancer by regulating the expression of cyclin D1 and proliferating cell nuclear antigen (PCNA) [27] and decrease cell viability in human HCT-116 colon cancer cells in dose- and time-dependent manners [28].

Therefore, ATX exhibits an obvious anti-proliferative effect in cancers. Furthermore, several studies indicated that the normal cells were unaffected/less affected than cancer cells by ATX. For example, although ATX significantly inhibited the proliferation of CBRH-7919, SHZ-88 and Lewis cell lines, it had little effect on HL-7702, a normal human hepatocyte line [19], indicating differential effects of ATX and focused targeting of cancer cells.

### 2.2. Apoptosis

Apoptosis is the process of programmed cell death (PCD) that takes place in multicellular organisms and comprises of many cellular events including nuclear fragmentation, cellular blebbing, chromosomal DNA fragmentation and ultimately cell death [29,30]. In physiological state, apoptosis is carried out in a regulated process, conferring advantage during an organisms life cycle occur. However, if apoptosis occurs in tumor cells, the tumor volume would decline, thus diminishing tumor burden and raising life expectancy [31,32]. In this regard, the effect of ATX on apoptosis is of interest and has been studied by researchers. The results obtained by Song *et al.* [19] showed that a significant peak of hypodiploid indicative of apoptosis was detected by flow cytometry when the cells were treated with ATX. Moreover, ATX caused changes in mitochondria morphology, transmembrane potential and respiratory chain and regulated apoptotic proteins in mitochondria such as B-cell lymphoma 2 (Bcl-2) and Bcl-2-associated X protein (Bax). In a hamster model of oral cancer, Kavitha *et al.* [14] reported that ATX could induce caspase-mediated mitochondrial apoptosis by down-regulating the expression of anti-apoptotic Bcl-2, p-Bcl-2-associated death promoter (Bad) and survivin and up-regulating pro-apoptotic Bax and Bad, accompanied by efflux of Smac/Diablo and cytochrome c into the cytosol and cleavage of poly (ADP-ribose) polymerase (PARP). In another study, ATX decreased the expression of Bcl-2, B-cell lymphoma-extra large (Bcl-xL) and c-myc while increased the level of Bax and non-metastasis23-1 (nm23-1) in a hepatocellular carcinoma cell line [20]. Taken together, these data suggests that ATX could induce mitochondria-mediated apoptosis in cancer cells.

Researches so far have only focused on the effect of ATX in mitochondria apoptosis pathway. However, depending on various cell death stimuli, apoptosis can be divided into intrinsic pathway (mitochondrial death pathway) and extrinsic pathway (death receptor pathway). The mitochondrial death pathway is controlled by members of the Bcl-2 family, including Bcl-2, Bad, Bax, Bid and Btf proteins on the mitochondrial membrane. Conversely, the death receptor pathway is mediated by Fas (CD95) and Fas-ligand [33,34]. Thus, whether ATX could induce extrinsic apoptosis remains unclear and further studies are needed to clarify it.

Interestingly, although ATX induced apoptosis in various cancers, it suppressed 6-OHDA-induced apoptosis and strikingly inhibited 6-OHDA-induced mitochondrial dysfunctions, including lowered membrane potential and the cleavage of caspase-9, caspase-3, poly(ADP-ribose) polymerase (PARP) in a human neuroblastoma cell line SH-SY5Y [5]. The discrepancies may be due to the complex and diverse interplays between ATX and apoptosis. Depending on different cell types, ATX may have different effects on apoptosis.

### 2.3. Anti-Oxidation

Oxidative stress is initiated by the production of free radicals and reactive oxygen species (ROS). Redox imbalance, due to aberrant ROS production and/or anti-oxidant functionality, contributes to tumor progression and is a hallmark of several types of cancer [35,36]. ROS may participate in cancer initiation, progression and spreading acting as secondary messengers in the activation and maintenance of specific signaling pathways [36].

This type of oxidative molecules can be inhibited by endogenous and exogenous anti-oxidants such as ATX. It has been shown that ATX attenuated intracellular O_2_^−^ production by restoring the anti-oxidant network activity of superoxide dismutase (SOD) and catalase (CAT), thus reversing lipopolysaccharide (LPS)-induced toxicity and ROS production in U937 cells [37]. In another case, ATX inhibited cell proliferation, induced cell apoptosis and interfered with cell cycle progression in leukemia K562 cells via activation of Nrf2-mediated anti-oxidant pathway [18]. Thus, oxidative stress could be key intermediates linking ATX and proliferation, apoptotic commitment.

However, recent studies have reported the pro-oxidant effects of some carotenoids on cancer cells with the generation of free radicals. Kim *et al.* [38] have observed the growth inhibition in leukemia cell lines by fucoxanthin and have attributed it to ROS generation by fucoxanthin that leads to apoptosis. Therefore, ATX may also exhibit its anti-cancer effects through activation of ROS. However, none studies have shown this action so far. Therefore, further studies are needed to clarify these mechanism.

### 2.4. Anti-Inflammation

The role of inflammation in the development of cancer was firstly described by Rudolf Virchow in 1863 [39]. Inflammation is part of the complex biological response of body tissues to harmful stimuli and is characterized by a general increase in plasma levels and cell capability to produce pro-inflammatory cytokines such as interleukin-6 (IL-6), interleukin-1 (IL-1) and tumor necrosis factor-α (TNF-α) [40,41]. This generalizes pro-inflammatory status, interacting with the genetic background and environmental factors, potentially triggers the onset of cancer [42,43]. Abundant evidence supports the preposition that various cancers are triggered by inflammatory disease [44,45,46] and anti-inflammatory drugs such as aspirin or cyclooxygenase-2 (COX-2) inhibitors could reduce tumor recurrence [47,48]. The effect of ATX on inflammation has also been explored in cancer. Speranza *et al.* [37,49] have reported that in U937 cell line, ATX inhibited ROS-induced activation of nuclear factor-κB (NF-κB) transcription factor, which then in turn effectively suppressed the production of inflammatory cytokines such as IL-1β, IL-6 and TNF-α, through a restoration of physiological levels of SHP-1. Furthermore, Yasui *et al.* [17] suggested that dietary ATX significantly inhibited the occurrence of colonic mucosal ulcers, dysplastic crypts and colonic adenocarcinoma which were related to colitis and colitis-related colon carcinogenesis in mice. They proposed that the suppression of inflammatory cytokines such as nuclear factor-κB (NF-κB), TNF-α, IL-1β, IL-6 and COX-2 contributed to the anti-cancer effect of ATX. Since inflammation affects all stages of cancer, for example, increasing the onset risk, starting the initial genetic mutation, supporting tumor progression and promoting invasion and metastasis, it could be the key target of ATX.

### 2.5. Invasion and Migration

Invasion and migration are two pivotal processes in the development of cancer [50]. To invade surrounding tissue and metastasize, malignant cancer cells break away from the primary tumor, attach to and degrade proteins that make up the surrounding extracellular matrix (ECM) [51]. Then cancer cells escape the original tumor site and migrate to other parts of the body via the lymphatic system, bloodstream or by direct extension [52]. In this process, matrix metalloproteinases (MMPs) play a crucial role. MMPs are zinc-binding endopeptidases that can promote tumor cell migration and invasion by breakdown of the ECM [53]. In contrast, tissue inhibitor of metalloproteinases (TIMPs) are the endogenous inhibitors of the zinc-dependent endopeptidases of the MMPs [54]. In a hamster model of oral cancer, Kowshik *et al.* [27] studied the effects of ATX on the expression of MMP-2 and MMP-9. These MMPs were overexpressed in cancer cells and degraded ECM during cancer invasion. ATX treatment resulted in decreased mRNA and protein levels of MMP-2 and MMP-9. Besides MMPs, they also studied TIMP-1 and reversion-inducing-cysteine-rich protein with kazal motifs (RECK), the endogenous inhibitors of MMPs. ATX increased the protein levels of TIMP-1 and RECK, suggesting the inhibition effects of ATX on invasion and metastasis. ATX was also found to suppress invasion in experimental rat colon carcinogenesis [16] and AH109A rat ascites hepatoma cell line [55] via modulating the expressions of MMPs. Thus, by inhibiting invasion factors, ATX may be valuable in preventing cancer cell invasion and metastasis.

### 2.6. Gap Junctional Intracellular Communication (GJIC)

GJIC is membrane structures made of intercellular channels that permits the diffusion of small hydrophilic molecules from cytoplasm to cytoplasm, resulting in metabolic and electrical coordination [56]. It regulates the communication between cells of an organ, allowing for direct communication between the cytoplasm of cells without transit through the extracellular space, making it possible for cells to achieve a common and integrated target/metabolic activity [57]. Gap junction (GJ) channels in vertebrate are formed by connexins (Cxs), a proteins family with at least 21 members in humans [58]. Each channel is formed by two hemichannels which are hexamers of homologous subunit proteins [56]. In general, loss of GJIC has been associated with pathologies such as cancer, cellular damage and inflammation [59]. Studies have suggested the enhancement of GJIC by ATX treatment. Hix *et al.* [60] reported that in a model of mouse embryonic fibroblast C3H/10T1/2 cell exposed to aqueous or aqueous/ethanol solutions, ATX up-regulated expression of Cx43 protein, increased formation of Cx43 immunoreactive plaques in regions of the plasma membrane consistent with localization of GJ and increased GJIC, which may result in inhibition of *in vitro* neoplastic transformation of 10T1/2 cells as well as growth reduction of human tumors in xenografts. Moreover, ATX inhibited methylcholanthrene-induced neoplastic transformation by up-regulating GJIC and increasing Cx43 protein expression [61]. In addition, Daubrawa *et al.*, have also observed increased GJIC in primary human fibroblasts in respond to ATX [62].

## 3. Molecular Targets of ATX in Cancers

**Figure 2:**
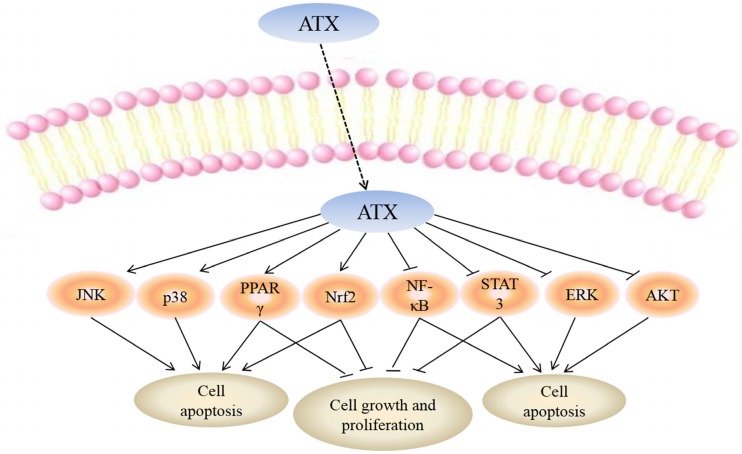
Molecular targets of ATX. Uptake of FTY720 into the cell leads to its direct activation of c-Jun *N*-terminal kinase (JNK), p38, peroxisome proliferator-activated receptor gamma (PPARγ) and NF-E2-related factor 2 (Nrf2) while inhibiting nuclear factor kappa-light-chain-enhancer of activated B cells (NF-κB), signal transducers and activators of transcription-3 (STAT-3), extracellular signal-regulated kinaes (ERK) and protein kinase B (PKB or AKT). Targeting these molecular targets may result in apoptosis of cancer cells. Moreover, activation of PPARγ, Nrf2 and inhibition of NF-κB, STAT-3 can suppress cell growth and proliferation.

While the mechanisms mediating the anti-cancer action of ATX have yet to be fully clarified, a number of molecular targets of ATX have been proposed which may explain the biological effects of this drug (Figure 2).

### 3.1. NF-κB

NF-κB comprises a family of transcription factors, which positively regulate the expression of genes involved in inflammatory and other responses by binding to their promoters [63,64]. In addition, researches have shown that NF-κB can control cell proliferation by inducing growth factors [65,66]. NF-κB also served as a positive regulator of cell cycle progression as it can activate cyclin D1 and c-myc [67,68]. Moreover, NF-κB inhibited PCD through regulating members of the Bcl-2 family [69]. NF-κB activity can also lead to increased angiogenesis and metastasis through up-regulation of chemokines, including vascular endothelial growth factor (VEGF), IL-8 and MMPs [70,71]. Therefore, NF-κB has become one of the most important target of cancer therapy and drugs that suppress the NF-κB pathway may be essential to treat cancer. Gupta *et al.* [72] reported that triterpenoids attenuated the expression of proteins associated with proliferation (cyclin D1), apoptosis (Bcl-2, Bcl-xL), invasion (MMP-9) and angiogenesis (VEGF), which were all regulated by NF-κB.

The effect of ATX on NF-κB has also been studied. Nagendraprabhu *et al.* [16] reported that ATX exhibited anti-cancer effects on DMH-induced rat colon carcinogenesis by inducing apoptosis and regulating the expressions of NF-κB. A selective COX-2 inhibitor, etoricoxib, was reported to reduce the expression of NF-κB protein and inhibit DMH-induced colon ACF in rats. Moreover, ATX inhibited NF-κB and Wnt signaling by downregulating the key regulatory enzymes IKKβ and GSK-3β, leading to caspase-mediated mitochondrial apoptosis [14]. In addition, NF-κB signal pathway played an important role in the colitis-associated colon carcinogenesis and may be a potential target of colitis-related colon carcinogenesis [17].

The mediators between ATX and NF-κB have not been fully clarified, but recent literature indicated that ROS may be involved in the inactivation of NF-κB. For example, Gambogic acid induced oxidative stress dependent apoptosis and autophagy in bladder cancer cell lines by abrogating NF-κB activation. The inactivation effects of Gambogic acid on NF-κB was through ROS-mediated inhibition of IκB-α phosphorylation [73]. Thus, ATX may also regulate NF-κB through ROS, but to date, how ATX mediates NF-κB has not been explained. Although the relationship between ATX and NF-κB remains unclear, NF-κB may be a pivotal target of ATX in cancer therapy.

### 3.2. Janus Kinase/Signal Transducers and Activators of Transcription-3 (JAK/STAT-3)

The JAK/STAT signaling pathway is a pleiotropic cascade used to transduce a multitude of signals for development and homeostasis in animals [74]. Signaling through the JAK/STAT pathway could lead to cell proliferation, invasion and differentiation [75,76], resulting in pathological diseases such as cancer [77], allergy [78], renal disease [79] and hepatic disease [80,81]. The STAT proteins family contains 7 members including STATs 1, 2, 3, 4, 5A, 5B and 6 [82]. Of these proteins, the constitutive activation of STAT-3 is usually found in cancer cells, including multiple myeloma [83], leukemia [84] and prostate cancer [85]. STAT-3 is activated by phosphorylation through JAK and the activation of STAT-3 is a critical step in the apoptosis pathway.

Kowshik *et al.* [27] have attempted to elucidate the mechanism of the anti-proliferative, invasion and angiogenesis action of ATX by studying the JAK-2/STAT-3 signaling pathway. They found that ATX inhibited key events in JAK-2/STAT-3 signaling especially STAT-3 phosphorylation and subsequent nuclear translocation of STAT-3, leading to down-regulated of STAT-3 target genes involved in cell proliferation (cyclin D1, PCNA), invasion and angiogenesis (MMP-2, MMP-9) and angiogenesis (VEGF, VEGFR2), resulting in inhibition of tumor development and progression. Song *et al.* [20] have also observed that ATX regulated apoptotic protein like Bcl-2, Bcl-xL, c-myc and Bax via suppression of JAK-1/STAT-3 signaling pathway.

Besides JAK/STAT-3, there are several other forms of JAKs-STATs, including JAK-1/3-STAT-6, JAK-1/2-STAT-1/3/5 and so on. Recent data have also indicated a role of JAK-3/STAT [86] and JAK/STAT-5 [87] in the pathogenesis of cancer. For example, Gallipoli *et al.* [88] reported that combination of nilotinib with ruxolitinib could induce apoptosis in chronic myeloid leukemia (CML) stem cells through inhibiting JAK-2/STAT-5 signaling pathway. However, no reports have studied the effects of ATX on these forms of JAKs-STATs and, thus, remains to be explored.

### 3.3. PI3K/AKT

PI3K/AKT is an important intracellular signaling pathway in regulating cell survival and death. Signaling through this pathway controls proliferation and apoptosis of cells [89]. The phosphorylation of AKT can activate the mammalian target of rapamycin (mTOR) and further trigger the phosphorylation of the downstream target p70S6K, enhances the transcription of certain mRNAs, and increases the expression of proteins associated with proliferation [90,91]. Dysregulation of the PI3K/AKT signaling pathway has been reported in several types of cancer, including colorectal cancer [92], breast cancer [93], and cholangiocarcinoma [94]. Therefore, some experimental cancer drugs aim to inhibit the signaling sequence at some point.

ATX could also facilitate PI3K/AKT signaling pathway to induce cell death. In oral cancer, ATX apparently decreased the phosphorylation of AKT, followed by a reduction of Bcl-2, p-Bad and survivin, a concomitant increase of Bax, Bad and cleaved PARP, resulting in significant apoptosis [14]. Furthermore, ATX induced p-AKT down-modulation in experimental rat colon carcinogenesis, which then led to cell apoptosis [16]. In addition, in human colon cancer cells, ATX also showed anti-cancer effects by inactivation of AKT [28].

### 3.4. MAPKs

MAPKs are serine/threonine-specific protein kinases belonging to the CDK/MAPK/GSK3/CLK (CMGC) kinase group [95]. They participant in many cellular processes including proliferation, apoptosis and differentiation. Furthermore, the role of MAPKs in cancer has been well established in several models, such as ER stress [96], mitochondrial dysfunction [97] and oxidative stress [98]. There are three members in the MAPKs family including extracellular regulated protein kinase (ERK), c-Jun *N*-terminal kinase (JNK) and p38, among which the ERK and JNK are the most important in regulating cell death and survival.

It has been shown that in HCT-116 colon cancer cells, ATX inhibited cell growth in dose- and time-dependent manners by arresting cell cycle progression and promoting apoptosis. Moreover, ATX also increased the phosphorylation of p38, JNK and ERK, suggesting that ERK may promote apoptosis in this situation [28]. Interestingly, in a rat colon carcinogenesis model [16] and a hamster oral cancer model [14], ATX exerted anti-tumor efficacy through inactivation of ERK, indicating a protective effect of ERK.

The role of ERK in cancer is controversial. Zhang *et al.* [99] observed that overexpression of Annexin A1 (ANXA1) induced by arsenic trioxide (ATO) resulted in activation of ERK, rendering cancer cells resistant to the agent. In addition, PD98059, a specific ERK inhibitor, increased the sensitivity of cancer cells to ATO treatment, suggesting a protective role of ERK. However, in the study conducted by Baek *et al.* [100], they found that Cinobufagin (CBG) increased sub-G1 DNA contents of cell cycle, cleaved caspase-3 and PARP and caused the activation of ERK in multiple myeloma (MM) cells. The ERK inhibitor (PD98059) significantly prevented the CBG-induced caspase-3 and PARP cleavage, indicating that ERK promoted apoptosis. The discrepancies may be due to the complex and diverse interplays between ATX and ERK. Depending on cell types, environment and stimulus, ATX may have inhibitory or promoter action on ERK.

### 3.5. Peroxisome Proliferator-Activated Receptor Gamma (PPARγ)

PPARγ is a ligand-dependent transcription factor that belongs to the super family of nuclear hormone receptors. PPARγ is expressed principally in fatty and vascular tissue; however, it also presents in heart and brain tissue [101]. In particular, PPARγ plays an important role in the regulation of adipogenesis, lipid homeostasis and in the development of various organs [102]. Apart from the established metabolic actions, PPARγ also plays a key role in multiple types of cancer, including lung, colon, breast, prostate, pancreas and bladder. The activation of PPARγ plays an inhibitory role in cellular proliferation and growth, this property makes PPARγ an important target for the development of new drugs aimed at preventing and treating cancer [103]. In vitro and in vivo studies have demonstrated anti-proliferative and pro-apoptotic actions of PPARγ agonists such as 15-deoxy-∆-12,14-prostaglandin J_2_ (15dPG-J_2_) and thiazolidinediones (TZDs) [104,105], suggesting that PPAR*γ* could be a promising target for cancer therapy.

Recent studies have shed further insight into the mode of action of ATX by demonstrating that it directly up-regulated PPAR*γ*. Zhang *et al.* [18] have indicated that ATX could inhibit proliferation, decrease viability, induce apoptosis and interfere with cell cycle progression of leukemia K562 cells by increasing the expression of PPAR*γ*. Pretreatment with GW9662, a potent antagonist of PPAR*γ*, partly attenuated the inhibition of K562 cell proliferation by ATX.

The family of PPARs is mostly composed of three known isoforms: PPARα, PPARβ/δ and PPARγ. These receptors share a structural homology that consists of four functional units (A, B, C and D). In addition to PPARα, PPARβ*/*δ and PPARγ were also shown to be involved in cancer. Schumann *et al.* [106] reported that deregulation of PPARβ/δ target genes by ligands of the tumor microenvironment could contribute to the pro-tumorigenic polarization of ovarian carcinoma tumor-associated macrophages (TAMs). In another study, Zhang *et al.* [107] found that PPARα suppressed tumor cell growth by inhibiting cell proliferation and inducing cell apoptosis via direct targeting IκBα and NF-κB signaling pathway. However, no reports have studied the effects of ATX on PPARβ*/*δ or PPARγ. Thus, this is an interesting aspect worth exploring.

### 3.6. NF-E2-Related Factor 2 (Nrf2)

The transcription factor Nrf2 was initially regarded as a crucial regulator of intracellular antioxidants and phase II detoxification enzymes. Oxidative and redox stress activates Nrf2 and its downstream factors such as heme oxygenase-1 (HO-1), NAD(P)H dehydrogenase [quinone] 1 (NQO-1) and glutamate-cysteine ligase catalytic (GCLC), leading to the decreased reactive oxygen species (ROS) [108,109]. A number of studies have now shown that Nrf2 can protect cells in normal tissues from harmful stimulus, including cancer, trauma, inflammation and hemorrhage [110,111,112,113] and administration of Nrf2-inducing agents has been shown to result in decreased carcinogenesis in animal models and altered carcinogen metabolism in humans [114].

As yet, there have been two studies reporting the effects of ATX on Nrf2. Zhang *et al.* [18] demonstrated that ATX increased obvious Nrf2 expression, finally inhibiting K562 leukemia cell proliferation [18], suggesting that Nrf2 suppressed the progress of cancer. Interestingly, recent findings proposed that Nrf2 might also play a dark role in tumors. Reports have shown that constitutively high levels of Nrf2 promoted cancer formation and contributed to chemoresistance [115,116,117]. Further investigation demonstrated that Nrf2 was associated with cell proliferation by regulation of multiple signaling pathways [118,119]. Thus, in the experiment conducted by Speranza *et al.* [37], they found that ATX attenuated LPS-induced inflammatory and oxidative by inhibiting the activation of Nrf2 in U937 cells, indicating a protective role of Nrf2 in cancer. So, depending on different chemotherapeutics and cancer cell types, Nrf2 may has different roles. ATX although activated Nrf2 in leukemia cell, it may also suppress Nrf2 to achieve anti-cancer activity in other cancer types. It is worth noting that in the experiment conducted by Zhang *et al.*, they examined the total expression of Nrf2. While Speranza *et al.*, tested the nuclear and cytoplasm expression of Nrf2 respectively, they found that ATX inhibited nuclear translocation of Nrf2 but increased the cytoplasm expression of Nrf2. Therefore, although the total expression of Nrf2 increased in K562 cells in respond to ATX, its distribution in nuclear and cytoplasm was unclear. Further studies are needed to elucidate the influence of ATX on Nrf2 in cancers.

## 4. Absorption and Tissue Distribution of ATX

As a fat soluble compound, ATX also follows the same intestinal absorption path as dietary fat. Absorption of ATX is affected by the same factors that influence fat absorption. Thus, dietary oils could enhance the absorption [120] while the absence of bile or any generalized malfunction of the lipid absorption system will interfere with the absorption [121]. ATX mixes with bile acid after ingestion and make micelles in the intestinum tenue. The micelles with ATX are partially absorbed by intestinal mucosal cells. Intestinal mucosal cells incorporate ATX into chylomicra. Chylomicra with ATX are digested by lipoprotein lipase after releasing into the lymph within the systemic circulation, and chylomicron remnants are rapidly removed by the liver [122]. The liver does not convert chylomicron to vitamin A or otherwise biochemically transform it [123]. Instead it becomes incorporated into low-density lipoprotein (LDL) and high-density lipoprotein (HDL), which then distribute it to the tissues via the circulation [124]. When ATX is fed to human subjects, detailed pharmacokinetic data are difficult to obtain for single doses of less than 10 mg, due to limitations of assay precision. However, there is good data to indicate a single 10 mg dose can persist in the blood for 24 h and a 100 mg dose for 76 h. Doses as low as 1 mg can significantly increase blood levels when taken once daily for four weeks [125].

## 5. Future Prospects of ATX

Cancer is a broad group of diseases involving many characteristics. The biological properties of cancer include apoptosis, necrosis, autophagy, invasion and so on. Although the effects of ATX on proliferation, apoptosis, inflammation, invasion and migration has been widely described, its role in autophagy and angiogenesis have not been fully explained so far.

### 5.1. Autophagy

Autophagy is a process by which cells conserve and recycle their organelles when in a nutrient-deprived or stressed state [126]. During autophagy, targeted cytosolic proteins and organelles are isolated within the autophagosomes, which are then fused with lysosomes, the contents of the autophagosome are degraded via acidic lysosomal hydrolases [127]. Under physiological conditions, autophagy ensure cellular survival by maintaining cellular energy levels [128,129]. However, extensive or inappropriate activation of autophagy can lead to cell death (type II PCD). Nowadays, the relationship between autophagy and apoptosis is a hot research point in cancer. Recent studies have shown that some chemotherapeutics known to induce apoptosis also activate autophagy. However, depending on different stimulus and cell types, autophagy acts not only as a protector—it prevents cells from undergoing apoptosis [130] but also as a promoter—it promotes cell apoptosis [131]. Therefore, autophagy may be considered as a double-edged sword in cancer. Depending on cell types, environment and stimulation manners, autophagy and apoptosis may have inhibitory, additive or even synergistic effects.

There were also studies demonstrating that ATX could affect autophagy. Shen *et al.* [132] reported that ATX significantly improved the pathological lesions of liver fibrosis by decreasing the levels of alanine aminotransferase aspartate aminotransferase and hydroxyproline. Moreover, they found that the protective effect of ATX on liver fibrosis was through down-regulation of energy production in hepatic stellate cells (HSCs) by autophagy. In another study conducted by Li *et al.* [133], they observed decreased immune liver injury in concanavalin A (ConA)-induced autoimmune hepatitis by ATX. And this mode of action appeared to be down-regulation of JNK/p-JNK-mediated apoptosis and autophagy. Since autophagy played a key role in cancer and ATX has been shown to affect autophagy in liver injury model, therefore further studies are needed to estimate whether ATX could regulate autophagy in cancer.

### 5.2. Angiogenesis

Angiogenesis is the physiological process through which new blood vessels form from pre-existing vessels. Angiogenesis is a crucial part of tumor growth [134]. When a tumor reaches approximately 1–2 mm in diameter, it requires neovascularization for further development [135]. In addition, angiogenesis is a fundamental step in the invasion and metastasis of tumors. Therefore, disruption of tumor angiogenesis has been researched for developing alternative anti-tumor strategies. A number of studies have emphasized the major role of angiogenesis in cancer and agents that inhibited neovascularization could suppress the development of tumor [136,137]. To date, antibodies targeting the VEGF, such as bevacizumab, have proved therapeutically viable [138].

However, the role of ATX played in tumor angiogenesis has not been fully understood. Recently, Kowshik *et al.* [27] found that ATX significantly modulated the expression of VEGF, VEGFR2 and decreased HIF-1a nuclear translocation, resulting in decreased number of vessels in oral cancer. This study indicated the anti-angiogenic potential of ATX, which may provide a novel research idea for the treatment of ATX in other cancers.

## 6. Concluding Remarks

A growing number of studies show that ATX emerges as a key player in cancer therapy. It also influences a multitude of molecular and cellular processes. In this review, we have described the effects of ATX on cancer as well as some molecular targets of ATX involved in cancer-associated processes (such as apoptosis and inflammation). These observations make ATX an attractive therapeutic agent for developing novel treatment protocols, and possibly for combining with other chemotherapeutics to overcome drug resistance and achieve better outcomes. It is clear that further studies are required to elucidate the full spectrum of direct and downstream cellular targets of ATX. Ultimately, ATX may hold promise for clinical cancer therapy.
